# Nodular lymphocyte-predominant Hodgkin lymphoma characteristics, management of primary and relapsed/refractory disease and outcome analysis: the first comprehensive report from the Middle East

**DOI:** 10.1186/s12885-021-08074-2

**Published:** 2021-04-01

**Authors:** Saad Akhtar, M. Shahzad Rauf, Yasser Khafaga, Amani Al-Kofide, Tusneem Ahmed M. Elhassan, Mahmoud A. Elshenawy, Juzer Nadri, Ali Hassan Mushtaq, Nasir Bakshi, Mohammed Shamayel, Suleiman Al-Sweedan, Sohail Sarwar, Irfan Maghfoor

**Affiliations:** 1 Oncology Center, King Faisal Specialist Hospital and Research Center, Riyadh, Kingdom of Saudi Arabia; 2 Department of Pediatric Hematology/Oncology, King Faisal Specialist Hospital and Research Center, Riyadh, Kingdom of Saudi Arabia; 3 AlFaisal University, Riyadh, Kingdom of Saudi Arabia; 4 Pathology and Laboratory Medicine, King Faisal Specialist Hospital and Research Center, Riyadh, Kingdom of Saudi Arabia

## Abstract

**Background:**

Nodular lymphocyte-predominant Hodgkin lymphoma (NLPHL) is an uncommon variant of Hodgkin lymphoma. There is limited data on treatment, management of refractory and relapsed disease, and long-term outcome. Many registries or country-wide data reports are unable to provide detailed primary and subsequent management. We are reporting our observation on patient’s characteristics, management, and outcome.

**Methods:**

This single-institution retrospective cohort analysis includes NLPHL patients seen from 1998 to July 2019. We used Fisher’s exact test, chi-square, and Kaplan-Meier (KM) method for various analyses.

**Results:**

Two hundred patients were identified, (6.34% of all the HL). Male:female was 3:1. The median age at diagnosis was 22 years (4–79 years). Stage I-II in 145 (72.5%) cases. One hundred patients (50%) received chemotherapy, 68 (34%) chemotherapy + radiation therapy (RT); 87% of all chemotherapy was ABVD (adriamycin, bleomycin, vinblastine, dacarbazine). Thirteen patients (6.5%) received RT alone and 16 (8%) had surgery alone. Complete response in 82%, partial response in 5.5% and progressive disease in 10.5%. The median follow is 60 months (5–246). Median 5 and 10 years overall survival (OS) is 94.8 and 92.4% (stages I-II, 97.7 and 97.7%, stage III-IV, 94.8 and 92.4%). Median event-free survival (EFS) is 62.3 and 54% respectively (stage I-II, 72 and 64%, stage III-IV, 36.4 and 18.2%). Stage I-II vs III-IV OS (*p* = < 0.001) and EFS (*p* = < 0.001) were significant. For stage I-II, 5 year EFS of chemotherapy + RT (83.3%) was superior to chemotherapy alone (60%, *p* = 0.008). Five year EFS for early favorable (80%), early unfavorable (60%), and advanced (36.4%) was significant (*p* = < 0.001). Eleven patients (5.5%) had high-grade transformation. Twenty-nine patients underwent HDC auto-SCT, all are alive (28 in remission). 25% of patients had pathologically proved nodal hyperplasia at some point in time.

**Conclusion:**

OS of NLPHL is excellent and independent of treatment type. EFS is better for chemotherapy + RT than chemotherapy alone. Stem cell transplant in refractory / multiple relapses resulted in excellent disease control. There is a need to identify optimal treatment strategies accordingly to the risk stratification.

## Background

Nodular lymphocyte-predominant Hodgkin lymphoma (NLPHL) is a relatively uncommon subtype of Hodgkin lymphoma (HL) that accounts for about 5–6% of all HL cases. World Health Organization classification of lymphoid neoplasm recognizes NLPHL as a separate entity with unique morphologic, immunohistochemical, and clinicopathological characteristics [1]. The presence of atypical “lymphocyte-predominant cells”, (LP cells) or popcorn cells is characteristic. These cells express CD20 positivity and CD 15 and CD 30 negativity in a background of non-malignant and reactive nodular small mature B-lymphocytes which is a characteristic histological feature of NLPHL [2]. Although long-term survival is better than classical HL, frequent relapses and progression/transformation to aggressive non-Hodgkin lymphoma (NHL)/ diffuse large B cell lymphoma (DLBCL) is reported [3–8]. There is no randomized trial addressing the treatment strategies specifically designed for NLPHL and most of the data and information are extracted from either small series or subset analyses of larger HL registries. Data are even limited for relapsed and refractory NLPHL. There are few reports on high dose chemotherapy and auto-SCT (HDC auto-SCT) in relapsed and refractory setting [9–12]. Due to the expression of CD20 positive cells, rituximab has been reported as an effective treatment in NLPHL [13, 14]. Studies are reporting a higher incidence of both solid and hematological malignancies in the families of these patients. We are reporting the first large data from the Middle East on patient characteristics, treatment, and outcome in a relatively uniformly treated group of younger patients with long follow-up. We also discuss the finding of reactive lymphadenopathy in these patients during their disease, high-grade transformation (HGT), and its management and outcome. In this report, we will also update the outcome of our previously reported patients who underwent HDC auto-SCT.

## Methods

### Study design and ethical permission

This is a single institution retrospective cohort analysis using Institutional Research Advisory Counsel and Ethics Committee approved adults (age > 14 years) and pediatrics (age < 14 years) lymphoma databases. This waiver of signed consent was approved by the Research Advisory Council for participation in the database collection study and subsequent publications as per. Verbal consent was obtained and documented in the paper/electronic chart. All patients and/or their guardians provided informed consent for all treatments and procedures as per institutional requirements.

### Patients and definitions

All NLPHL adult and pediatric patients seen in the hospital from 1998 to July 2019 were identified using Adult Medical Oncology and Pediatrics Hematology / Oncology lymphoma databases. Patients with a concomitant diagnosis of NLPHL and transformed DLBCL were not eligible. Paper and electronic charts were reviewed to collect patient’s characteristics, treatment details, follow up and outcome data. Ann Arbor / Cotswolds modification staging system was used for staging. The refractory disease was defined as partial response (PR), no response (NR)/ stable disease (SD), or progressive disease (PD) after planned chemotherapy with or without radiation therapy (RT) or relapsing within 3 months from the end of the planned treatment after achieving a complete remission (CR) or CR unconfirmed (CRu). RT details were collected by a senior RT physician (YK) and included RT site, total dose delivered, dose per fraction, field information i.e. extended field RT (EFRT), involved field RT (IFRT), and involved site RT (ISRT). Information on RT treatment technique i.e. conventional 2D, 3D-conformal, or volumetric modulated arc therapy (VMAT) intensity-modulated RT (IMRT), and RapidArc were also collected.

### Imaging studies and response assessment

All patients had staging CT scans of chest, abdomen, and pelvis with and without contrast and CT scan of the head, neck, and other areas as needed. Many patients had 18F-fluorodeoxyglucose positron emission tomography (FDG-PET/CT) scan at staging and response assessment as it became available. CR, PR, NR/SD, PD were all defined according to the International Working Group [15] response assessment/criteria for CT scan. Mediastinal blood pool structure uptake activity and subsequently Deauville five-point criteria was used for FDG-PET/CT scan interpretation [16].

### Pathology review

Expert hematopathologist/s reviewed the pathology specimens. All these reports were re-reviewed by two hematopathologists (NB, MS) and specimens (as needed) were reevaluated until complete agreement achieved in all cases as needed. The World Health Organization (WHO) 2008 criterion for diagnosing lymphoma was used [17]. All available pathology reports were reviewed to identify reactive lymphadenopathy.

### Statistical analysis

Patient characteristics were summarized using frequencies and medians with ranges and compared using Fisher’s exact test and Mann-Whitney U test for categorical and continuous variables respectively. Survival probabilities were calculated using Kaplan-Meier (KM) methods with variance estimated using the Greenwood formula. Overall survival (OS) was defined as time to death from any cause and event-free survival (EFS) was defined as time to persistent, progressive, relapsed disease or death. Patients who are alive and disease-free at the time of the last follow-up are censored. Cox proportional hazard model was utilized to compare the risk between study groups. All variables were tested for affirmation of proportionality assumption and no variable had violated the assumption. All statistical analysis was carried out using SPSS version 23 and *P*-value < 0.05 was considered significant. However, for subgroup analysis, a *p*-value less than 0.01 was considered significant to adjust for multiple comparisons. We also risk-stratified our patients according to the German Hodgkin Study Group (GHSG) risk models; early favorable, early unfavorable, and advanced [18]. “KM survival analysis was also performed for different prognostic factors and clinical and laboratory parameters. Factors with a p-value < 0.1 were tested for multivariate analysis; we used Forward Method utilizing the Likelihood Ration (LR) with the entry criteria 0.1 and 0.05 for the removal.

## Results

### Patients characteristics

Between 1998 to 7–2019, we identified 200 patients with NLPHL who met the eligibility criteria. Male:female is 3:1. The median age at diagnosis is 22 years (4–79 years) with 98 (49%) patients < 21 years at diagnosis. The median age was similar for both females (22.4 years) and males (21 years). Our hospital tumor registry is updated till 2014, from which we identified a total of 2020 patients of HL from 1998 to 2014, of those, 128 were NLPHL, representing 6.34% of all the HL during this time frame. There is likely to be a higher percentage of NLPHL from 2015 to 2019 as 72 cases have already been identified. Further detailed analysis can only be performed once 2015–2019 data is available from the hospital tumor registry. Patient characteristics at diagnosis, their treatment, and the outcome are shown in Tables 1 and 2. All patients had conventional CT scanning for staging. During 2010–2019, staging with FDG-PET/CT scan became increasingly common. From 1998 to 2009, 27/85 patients (31.8%) had staging FDG-PET/CT scan as compared to 99/115 patients (86%) from 2010 to 2019 (p = < 0.001). Despite this higher use of FDG-PET/CT scan, there was no change in the incidence of stage I-II (71.2 and 73%, *p* = 0.87) during these two periods respectively. Early stages represented the majority of the patient population; stage I-II in 145 (72.5%) cases. Only 3/145 patients (2%) with stage I-II had B symptoms as compared to 11/55 (20%) with stages III-IV (p = < 0.001). The bulky disease was present in 8/145 patients (5.5%) with stage I-II as compared to 11/55 (20.4%) with stages III-IV (*p* = 0.012). Laterality information for stage I-II was also available for 141/145 patients; 39 (28%) right-sided, 53 (37.6%) left-sided and bilateral in 48 (34%) patients, *p*-value not significant.

**Table 1:** Patient’s characteristics at initial presentation

Variable	Total patients	Percentage
Total patients	200	100
Male	151	75.5
Female	49	24.5
Median age at diagnosis	200	22 yrs. (4–79 years)
< 14 years	40	20
> 14–21	57	28.5
> 21–30	39	19.5
> 30–50	50	25
> 50	14	7
Stage I	75	37.5
Stage II	70	35
Stage III	41	20.5
Stage IV	14	7
Bulky disease involvement	19	9.5
Spleen involvement	22	11
Extranodal involvement	25	12.5
Bone marrow involvement	5	2.5
B symptoms present	14	**7**
Early favorable^a^	96	48
Early unfavorable^a^	14	7
Advanced^a^	55	27.5
Risk group unknown^a^	35	17.5
LDH > normal	40	20
LDH missing	50	25
Performance status > 2	6	3
Performance status unknown	44	22

^a^As per German Hodgkin Study Group

**Table 2:** Treatment and survival outcome

Variable	Total patients	Percentage
Initial treatment		
Chemotherapy	100	50
Radiotherapy^a^	13	6.5
Surgery alone	16	8
Chemotherapy + Radiotherapy	68	34
Rituximab alone	2	1
Refused / No show	1	0.5
Type of chemotherapy		
ABVD^b^	147	73.5
ABVD/ABV with COPP like	3	1.5
COPP^b^	5	2.5
R-CHOP^c^	11	5.5
Others^d^	6	3
Number of initial chemo cycle		
2 cycles ^a^	31	15.5
3 cycles	11	5.5
4 cycles	64	32
6 cycles ^a^	53	26.5
8 cycles	11	5.5
Response after initial treatment		
CR	164	82
PR	11	5.5
PD^b^	21	11.5
On treatment	1	0.5
Tissue confirmation at Rx failure		
Type of first event	200	100
No event	124	62
Persistent disease	12	6
Progressive disease	21	10.5
Relapsed disease	36	18
Treatment related mortality	1	0.5
Death other causes	1	0.5
Unknown	5	2.5
Disease status at last visit		
Alive in remission	176	88
Alive with disease	4	2
Alive unknown status	1	0.5
Lost to follow up	6	3
On treatment	2	1
Died of disease	8	4
Died of other cause^c^	3	1.5
High grade transformation	11	5.5
HDC auto-SCT	29	14.5

*Abbreviations*: *ABVD* Adriamycin, bleomycin, vinblastin and dacarbazine, COPP/MOPP Cyclophosphamide / mechlorethamine, vincristine, procarbazine, prednisone, *PR* partial response

^a^ Rituximab × 4 before /or after radiotherapy in 2 patient, ^b^ Rituximab + ABVD in 9, Rituximab + COPP in 3, ^c^ Rituximab + CVP in 2, ^d^ Rituximab alone (3), rituximab + gemcitabine (1), dexamethasone, Ara-C, cisplatin (1), unknown at other institution (1). ^a^ 1 cycle in 2, 5 cycles in 4, ^b^2 patients with no response (NR) or stable disease (SD) in this group, progressive disease (PD), complete remission (CR), complete remission unconfirmed (CRu), ^c^treatment related mortality in 2 (both with lung toxicity after R-ABVD, both in CR (1 for relapsed disease after radiation therapy). 1 patient with sickle cell related sever cardiomyopathy, died in CR

### Treatment

Details are shown in Table 2. One hundred sixty-eight patients (84%) received chemotherapy or chemotherapy + RT as the first line. ABVD (adriamycin, bleomycin, vinblastine, and dacarbazine) was the most common regimen in 146 (73%) of 200 patients (87% of all chemotherapy). Another 22 (11%) had other combinations, including 12 adriamycin-based regimens. 81 (40.5%) patients received RT, 68 (34%) with chemotherapy and 13 (6.5%) as RT alone.

Treatment of stages I-II patient was chemotherapy alone in 53 (36.5%), RT alone in 13 (9%) (2 patients also received rituximab), chemotherapy + RT in 62 (42.7%), surgery alone in 16 (11%) followed by observation and rituximab alone in 2 (1%) patients. Treatment of stages III-IV patients was chemotherapy alone in 47 (85.5%), chemotherapy + RT in 6 (11%), and unknown in 1.

Rituximab was not commonly used in the first-line treatment of NLPHL up until 2016. Rituximab was used in 28 (14%) patients; from 2005 to 2015, only 6/112 (5.4%) patients received rituximab as compared to 21/55 (38.2%) from 2016 to July 2019. Rituximab was used alone in 2, with radiation in 2, with chemotherapy and RT in 3, and with chemotherapy in 21 patients. Rituximab was combined with CHOP (cyclophosphamide, adriamycin, vincristine, prednisone) (R-CHOP) and ABVD in 9 patients each, with CVP (cyclophosphamide, adriamycin, vincristine) in 2 and with COPP (cyclophosphamide, vincristine, procarbazine, prednisone) in 3. In these patients, the stages were I-II in 12 and III-IV in 16. The Median follow-up of these patients is only 24 months. KM estimates of 24 months EFS is 75.3% (rituximab group) vs 74.5% in non-rituximab group (*p* = 0.7). Cox Regression for no-rituximab use was not inferior, (HR = 1.2, 95% CI = 0.5–2.8, *p* = 0.7). For stage III-IV alone, 16/55 (30%) received rituximab in the first line, 24 month KM EFS estimate is 66.5% (rituximab group) vs 55% (no rituximab group) (*p* = 0.42), median EFS was 30 months in each group. The numbers and follow-up are too small for any meaningful subgroup analysis and conclusion.

Radiation therapy

81/200 patients received RT during their initial treatment (one patient who refused radiation after 2 fractions have been excluded from all the analysis). RT was given both to males (44%) and females (31%), and this was not significant (*p* = 0.32). Almost 50% of patients with stages I (41/76 (53%)) and II (34/69 (49%)) received radiation. Another 6/41 (14.6%) stage III also received RT. The reason for RT was consolidation (already in CR) in 42 (52%), to eradicate residual disease after chemotherapy in 20 (25%), as a primary/first-line treatment in 13 (16%) and in 6 (7%), disease status after ABVD chemotherapy / before RT was not checked. Dose of radiation was 15 to 41.4 Gy; 15 to 25.5 in 25 (31%) and 30 to 41.4 in 56 (69%). There was no difference in the outcome between the two dose groups (*p* = 0.6). Only 2 patients (before 2005) received > 36 Gy. Patients who achieved CR on chemotherapy usually received a lower RT dose around 30.6 Gy (38/43). The field of RT was IFRT in 66 (81.5%), ISRT in 14 (17%), and extended field RT in 1 patient. There was no difference in the outcome between the two RT field groups (*p* = 0.5), although, numbers are too small for any analysis. Over the years, the RT treatment technique has evolved. It was conventional 2D in 11 (13%) patients, 3D-conformal in 42 (51%) patients and VMAT/IMRT in 29 (36%). There was a significant difference in the outcome of these patients due to the reason of RT.

5-year KM EFS for RT as consolidation was 97% vs as a primary/first-line treatment (63%, HR = 7, 95% CI = 1.3–36, *p* = 0.02) compared to RT as consolidation. 5-year KM EFS for eradicating residual disease after chemotherapy was 36%, HR = 19, 95% CI = 4.7–80) as compared to RT, *p* < 0.001.

### Response to treatment and survival analysis

**Fig. 1:**
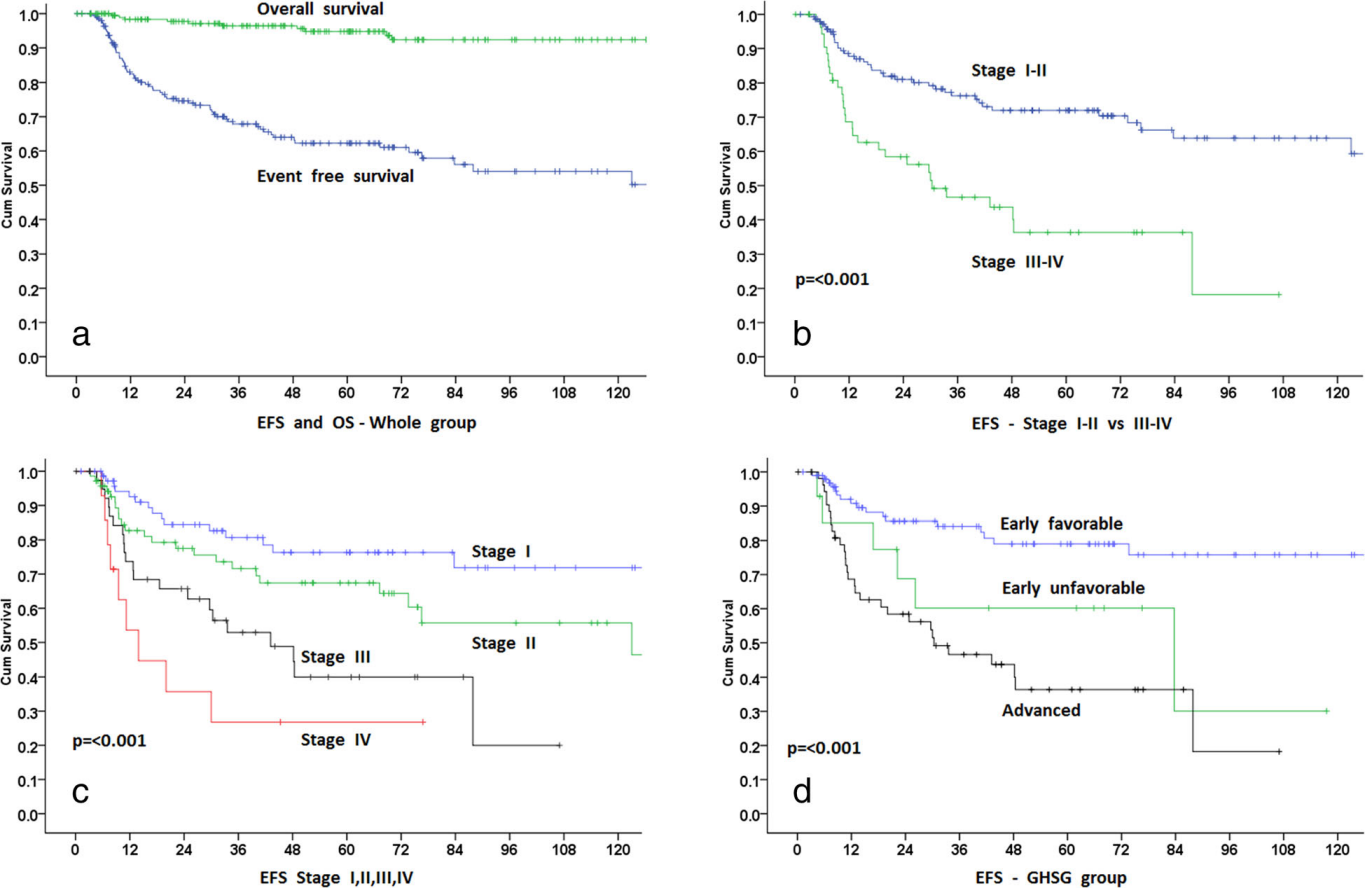
**a**, overall and event free survival of entire group. **b**, event free survival with stage I-II and stage III-IV. **c**, event free survival with stage I, II, III and IV. **d**, event free survival according to the German Hodgkin Study Group risk models; early favorable, early unfavorable and advanced

**Fig. 2:**
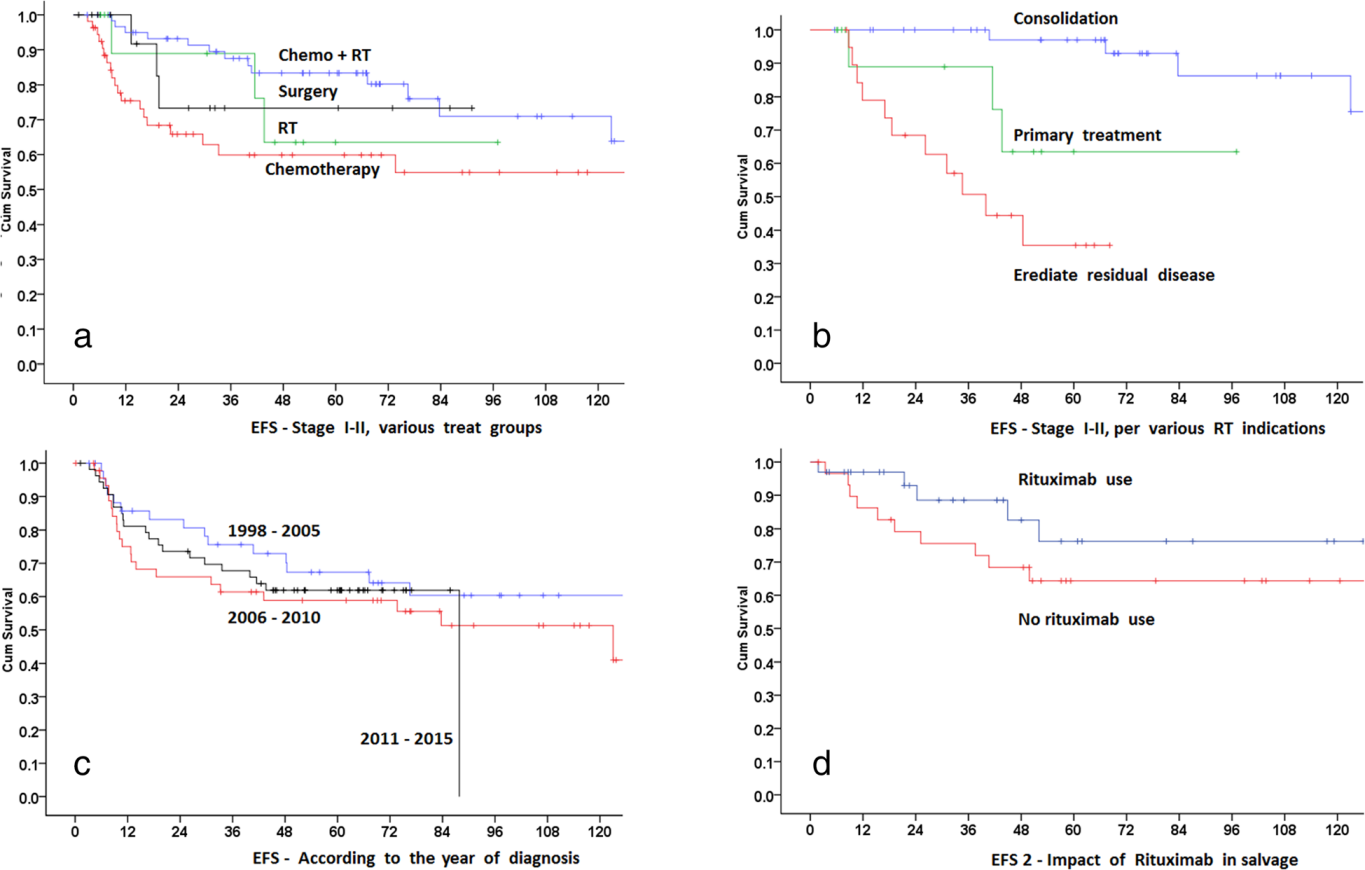
Impact of various factors on event free survival. **a** with chemotherapy + radiation therapy, surgery, radiation therapy alone and chemotherapy alone. **b** reason for radiation therapy (consolidation vs primary treatment vs eradication of residual disease). **c** various year-groups at the time of diagnosis. **d** rituximab in first line

**Fig. 3:**
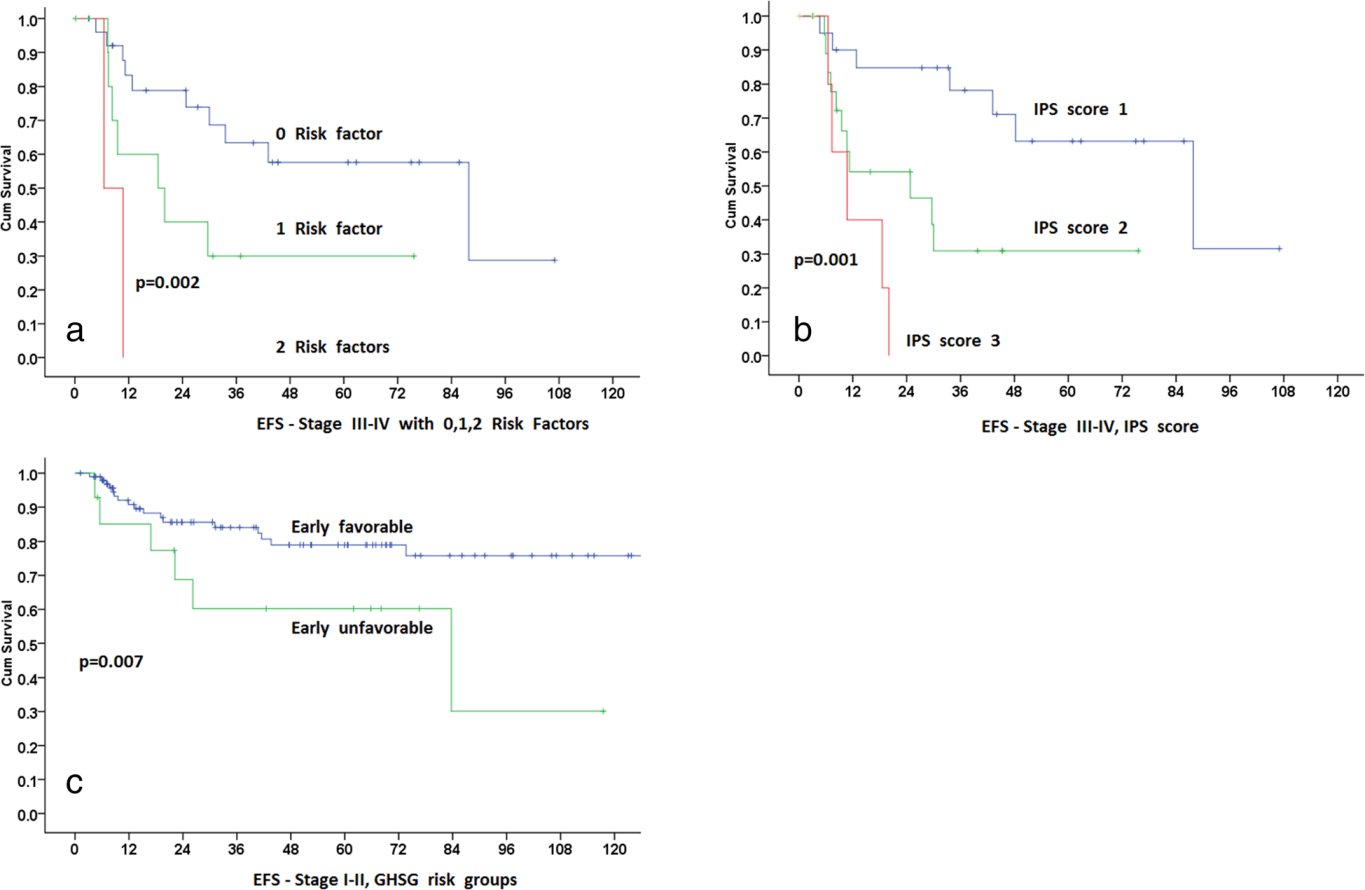
Impact of various factors identified on multivariate analysis and HL- International Prognostic Score on EFS. **a** EFS of stage III-IV with 0, 1, 2 risk factors. **b** EFS and HL- International Prognostic Score. **c** EFS of stage I-II according to the German Hodgkin Study Group risk models; early favorable and early unfavorable

The Median follow-up of all the alive patients is 60 months (1 to 246 months). Twenty-five alive patients (5 lost to follow-up) have less than 12 months follow-up. Overall response was CR in 164 (82%), PR in 11 (5.5%), PD in 21 (10.5%) cases (including 2 patients with no response) and unknown in 3 (1.5%). Median 5 and 10 years OS for the entire group is 94.8 and 92.4% respectively. The Median 5 and 10 year EFS for the entire group is 62.3 and 54%. As the treatment modalities and expected outcome are different, stages I-II and III-IV are analyzed separately for the types of treatment and outcome. These survival analyses are shown in Figs. 1, 2, and 3.

### Stage I-II

For stages I-II (145 patients), response to the treatment was CR in 129 (89%), PR in 5 (3.4%), PD in 9 (6.2%), and unknown/on treatment in 2 (1.4%). The response was also separately evaluated for all treatment groups. For chemotherapy (54 patients (2 patients with rituximab alone included in chemo group)), it was 77.8, 7.4, 11.1, and 3.8% respectively. For RT (13 patients), it was 100% CR, for chemotherapy + RT (62 patients), it was 93.5, 1.6, 4.8 and 0% respectively. For surgical excision and observation, it was 100% CR. For stage I-II, for various treatment modalities, only 5 year EFS of chemotherapy + RT (83.3%) was superior to chemotherapy alone (60%) (*p* = 0.008). For all other groups, the outcome was not statistically different. Cox regression also showed that chemotherapy alone was inferior to chemotherapy + RT (HR = 3, CI = 1.7–5.2, *p* = < 0.001). However, RT alone and surgery alone were not statistically inferior to chemotherapy + RT (HR = 1.41, CI = 0.14–4.8, *p* = 0.58) and (HR = 1.23, CI = 0.36–4.2, *p* = 0.74) respectively.

Median 5 and 10 years OS for stages I-II is 97.7% for both 5 and 10 years. A total of three stage I-II patients have died, one due to chemotherapy-related lung toxicity. KM estimates of 5 and 10 years EFS were 72 and 64% (stage I, 76.3 and 72%, stage II, 67.4 and 55.7% respectively). The Median KM estimate of EFS was 146.5 months (stage I, not reached, stage II, 123 months).

We also checked if there is a difference in the EFS based on CT scan staging (53/145 patients) vs FDG-PET/CT scan staging (92/145). Median KM estimates of EFS were 150 months (5 and 10 years EFS was 71 and 67%) for CT scan staging. Median KM estimates of EFS were 146.5 months (5 and 10 years EFS was 74 and 63%) for FDG-PET/CT scan staging, (*p* = 0.8) and showed that there was no EFS difference regarding staging methodology over time.

### Stage III-IV

CR was observed in 35 (64.8%), PR in 6 (11%), PD in 12 (22.2%) and unknown in 1 (2%). For chemotherapy alone (48 patients), it was 62.5%, 12.5, 22 and 2% respectively. For chemotherapy + RT (6 patients), it was 83.3, 0, 16.7 and 0% respectively. Median 5 and 10 years OS for stages III-IV was 87.6 and 79.5%. KM estimates of 5 and 9 years EFS were 36.4 and 18.2% (stage III, 40 and 20% and stage IV 26.8% and not available/short follow-up respectively). The median KM estimate of EFS was 30.4 months (stage III, 43 months, stage IV, 14 months). Cox regression was not performed due to small numbers in the chemotherapy + RT group. The difference in OS between stages I-II vs III-IV OS (*p* = 0.001) and EFS stages I-II vs III-IV EFS (*p* = < 0.001) was significant. EFS on CT scan staged patients (21/55 patients) vs FDG-PET/CT scan staged patients (34/55) was also checked. Median KM estimates of EFS were 30 months (5 years EFS was 45%) for CT scan staging. Median KM estimates of EFS were 30.4.5 months (5 years EFS was 25%) for FDG-PET/CT scan staging, (*p* = 0.57).

### GHSG risk models and outcome

According to GHSG risk stratification model, 34 patients (34/145 stage I-II (18/76 (23.7%) stage I) and (16/69 (23.2%) stage II)) had missing information; mainly ESR, and these cases were unclassifiable to early favorable or early unfavorable. Except for 1 stage I patient, 13 out of 14 early unfavorable patients had stage II. Progressive decline in the median EFS for early favorable (not reached), early unfavorable (83.7 months), and advanced (30.4 months) was significant (*p* = < 0.001). KM estimate of 5 and 10 years EFS for early favorable (80 and 77%), unfavorable stage (60 and 30%), and advanced stage (36.4 and 18% at 9 years) was significant (*p* = < 0.001). Compared to early favorable, the early unfavorable and advanced group had inferior EFS (HR = 2.7, CI = 1–6.8, *p* = 0.04) and (HR = 4.2, CI = 2.3–7.6, *p* = < 0.001) respectively.

### Reactive lymphadenopathy

Fifty (25%) patients had some form of pathologically proven reactive lymphadenopathy at some point in time. Biopsies done on clinical or radiologic suspicion of relapse/residual disease revealed 74 instances of reactive lymphadenopathy in 50 patients. Thirty-four patients had 1 biopsy specimen, 11 with 2, 3 with 3, 1 each with 4, and 5 biopsies showing reactive lymphadenopathy. Type of reactive lymphadenopathy was described in the pathology report as lymphoid hyperplasia (26 specimens), progressive transformation of germinal center (PTGC) (13 specimens), PTGC + hyperplasia (10 specimens), reactive node (24 specimens), and 1 patient as cattleman’s like picture. This reactive picture was noted concurrently in the primary NLPHL diagnostic specimen at the time of initial diagnosis in 18 patients and with the relapsed specimen in 3. In the remaining 53 specimens among 29 patients, when based on the clinical and/or radiological findings of a suspected relapse, a biopsy was obtained, only a reactive picture was the primary pathological finding. 5 and 10 year EFS for patients with no hyperplasia was 63 and 54% compared to 56 and 50% respectively in patients with hyperplasia (*p* = 0.99).

### High-grade transformation

Eleven patients (5.5%) developed transformation to an aggressive pathology after a median of 30 months (3–61 months) from the initial diagnosis. Pathology was T cell histiocytic rich B-cell lymphoma (a variant of DLBCL) in 6, DLBCL in 4, and classical Hodgkin lymphoma - lymphocyte rich variant in 1. Lactate dehydrogenase was elevated in 6, normal in 3, and not available in 2 patients. No other analysis was performed due to small numbers. Six patients had salvage therapy and HDC auto-SCT and another patient is in CR after salvage chemotherapy, awaiting allogeneic stem cell transplantation after failing stem cell collection. One patient with anthracycline-induced cardiomyopathy due to prior ABVD is in CR after salvage chemo. One patient refused all treatment at transformation and died 15 months after HGT. One patient was refractory to 3 lines of treatment and died 13.5 months after HGT. One patient had limited relapse in the spleen and is in CR for 10 years after splenectomy alone. Nine patients received rituximab in salvage and are alive in CR (17–150 months).

### Treatment failure and salvage treatment

Ninety-five events were observed in 71 patients (Table 3). Fifty-three patients had 1 event each, 12 had 2 events each, 3 with 3 events each, and two had 4 events each. These events were persistent disease 15 times, progressive disease 29 times, 48 relapses and 1 each TRM, and death due to other causes.

**Table 3:** Univariate and multivariate analysis of various prognostic factors

**Kaplan Meier 5 years EFS and OS** **Univariate Analysis**						
**Covariates / different groups**	**Stage I-II** **EFS**		**Stage III-IV** **EFS**		**Stage III-IV** **OS**	
	%	*p*-value	%	*p*-value	%	*p*-value
Female	71.4	0.57	21.4	0.82	91	0.74
Male	72.2		40		87	
Age < 21	69.2	0.56	48.4	0.151	95.5	0.71
Age > 21	75.1		26		81.6	
Age < 30	69	0.55	45.8	0.29	97	0.093
Age > 30	79.3		18.6		71	
Performance status 0–1	71.7	*	33.7	0.54	87	0.48
Performance status > 2	100		0		75	
B symptoms - No	71.6	*	34.4	0.98	88.4	0.86
B symptoms - Yes	100		57.1		87.5	
Hemoglobin > 10.5	77.7	0.9	50.5	0.011	86.8	0.13
Hemoglobin < 10.5	85.7		16.7		67	
Albumin > 4	80.2	0.047	53.8	0.005	92	0.061
Albumin < 4	60		20		70	
LDH not elevated	82.7	0.007	67.3	0.012	100	0.025
LDH elevated	61		19		75	
Bulky disease No	73	0.59	35.5	0.26	89.2	0.72
Bulky disease Yes	85.7		31.8		81	
Spleen involvement - No	72.2	*	38.6	0.058	89.2	0.8
Spleen involvement - Yes	100		27.1		84.4	
Extranodal involvement - No	72.4	0.72	34.7	0.34	87	0.51
Extranodal involvement - Yes	66.7		32.8		87.5	
Bone marrow positive	–	–	34.2	0.35	86.4	0.46
Bone marrow negative			0		100	
GHSG - Early favorable	79		–	–		
GHSG - Early Unfavorable	60.2	0.04				
Radiation given	79.2	0.03	50	0.27	75	0.91
Radiation not given	63.8		35		86	
**Multivariate Analysis**						
	HR**	95% CI**	HR	95% CI (range)	HR	95% CI (range)
GHSG Early favorable	1		–	–	–	-***
GHSG Early unfavorable	3.84	1.44 -10.25				
Hemoglobin > 10.5	–	–	1		–	–
Hemoglobin < 10.5			4.862	1.5-15.76		
Albumin > 4	–	–	1		–	–
Albumin < 4			4.359	1.492-12.74		

*All the cases censored; no statistics performed (in stage I-II, performance status > 2 in only 1 patient, B symptoms - Yes in 3 patients, spleen involvement in only 1 patient.). **Hazard ratio (HR), confidence interval, Lower - Upper limits (CI), other abbreviation as per Table 1. ***no factor showed significance on multivariate analysis

### Management of treatment failure

Eight of 71 patients had no further treatment; 5 of them had died, 1 TRM from first-line R-ABVD, 1 due to sickle cell-related cardiomyopathy, 3 refused any further treatment for relapsed (1 patient) and progressive (2 patients) disease. One patient relapsed in October 2016, refused any treatment, and is on observation and two patients are going through restaging workup (relapsed in November 2019).

#### First-line failure management

Sixty-three patients received further treatment. Forty-nine/63 patients (77.8%) received chemotherapy; 25/49 received rituximab-containing salvage combinations. Twenty patients had salvage chemotherapy followed by HDC auto-SCT. Radiotherapy alone in 5 (8%), surgery only in 2 (3%), and rituximab alone in 7 (11%). Overall, 52 patients (82.5%) went into CR.

#### Second-line failure management

Eighteen patients received treatment. Fifteen/18 patients (83.3%) received chemotherapy; 8/15 received rituximab-containing combinations. Eight patients had salvage chemotherapy followed by HDC auto-SCT. Radiotherapy alone in 2 (11%) and rituximab alone in 1 (5.5%). Overall, 12 patients (66.6%) went into CR.

#### Third line failure management

Three/5 (60%) received chemotherapy; all rituximab-containing combinations. One patient had salvage chemotherapy followed by HDC auto-SCT. Radiotherapy alone in 2 (40%). Three patients (60%) went into CR.

Overall, rituximab was used in the salvage setup in 42/63 patients 66.6% (33 in the first line salvage and another 11 in subsequent treatment failures).

#### HDC auto-SCT

Twenty-nine patients underwent HDC auto-SCT. As stated above, 20 at 1st, 8 at 2nd, and 1 at 3rd failure. 23/29 had carmustine, etoposide, cytarabine, and melphalan (BEAM) as HDC. CR was observed in 27 (93%) patients and PR in 2 (7%). 4 patients observed treatment failures. Two patients relapsed; one went into CR with rituximab alone and the other received 3 more lines including rituximab maintenance for 24 months and is in CR. One PR patient went into CR after radiation and one PR was observed for 9 years for slowly progressing disease, had salvage chemo, and progressed again. He is on observation again. All 29 patients are alive, and 28 patients are in CR. 20/29 (69%) patients received rituximab at some point in time. The most commonly used salvage was ESHAP (etoposide, solumedrol, Ara-C, and cisplatin) in 22/29 patients.

### Impact of various prognostic factors on the outcome

We evaluated different prognostic, clinical, and laboratory factors and also performed multivariate analysis as shown in Table 3 and Fig. 3. Gender has no impact on EFS; EFS at 5 years for males (62%) and females (59%), (HR = 1.17, CI = 0.67–2, *p* = 0.56).

We divided the age at diagnosis into various groups and evaluated its impact on EFS. There was no difference in the type of treatment for these groups (*p* = 0.9). EFS at 5 years for patients age < 21 vs > 21 years (*p* = 0.52) or < 30 or > 30 years (*p* = 0.72) was similar. For patients age < 14 years (52.5%), > 14–21 (73.7%), > 21–30 (59%), 30–50 (61.6.4%) and for > 50 (53%) failed to show a statistically significant trend (*p* = 0.22, Log-Rank method). Although, Pairwise analysis did show that age group 14–21 has superior EFS compared to < 14 group (*p* = 0.016) but not with other groups. Cox Regression for age group 14–21 favored better outcome compared to age < 14, (HR = 0.44, 95% CI = 0.22–0.88, *p* = 0.02). For ages 21–30 (HR = 0.5), 30–50 (HR = 0.4) and age > 50 (HR = 0.62) was not significant.

We also analyzed the impact of the year of diagnosis. Patients diagnosed in 2016 onwards were excluded as they had a significantly shorter follow-up. One hundred forty-five patients (1998 to 2015) were divided into three groups according to the year of diagnosis. From 1998 to 2015, treatment was similar without rituximab (only 6 received rituximab as the first line during this time). Five year EFS according to the year of diagnosis group was similar for 1998–2004 (64%), 2005–2009 (59%) and 2010–2015 (62%), (*p* = 0.33).

Multivariate analysis for stage I-II identified GHSG risk group model of early favorable and early unfavorable (*p* = 0.007) as the most significant risk factor (Fig. 3c). For stage III-IV, albumin and hemoglobin levels (p = 0.007) were significant, and based on these two factors, we divided patients into 0,1 and 2 risk groups for EFS (Fig. 3a). We also plotted EFS for stages III-IV based on HL- International Prognostic Score (Fig. 3b) that showed significance too (*p* = 0.001). This variable cannot be included in the multivariate analysis as the International Prognostic Score scoring incorporated many factors already included in univariate analysis. The cures of these two models (Fig. 3a and b) are very similar.

## Discussion

Despite the rarity of the condition, we believe, we are reporting the largest single-institution data to date. Our data includes detailed patient characteristics, risk factors, primary and subsequent salvage treatments including HDC auto-SCT, but also an important observation of reactive lymphadenopathy. There are limited studies in the last 20 years, reporting around 100 or more patients [3–8, 18, 19], mostly from various national registries, pooled multi-institutional data, or subgroup analysis from cooperative HL trials. These studies have provided large data with varying details on patient characteristics, treatments, and outcomes. We have included these large studies in Table 4 as a reference, only the most recent study is included in case of repeat/updated reports.

**Table 4:** Literature review of large NLPHL studies

Study (Publication year)	Study Design (years of treatment)	Numbers	Stage / population	Modality of Treatment	Median Follow up	PFS / EFS	OS	Rx for failure or 2nd line Rx	Median age (range)	Comments
Diehl et al. 1999 [20]	Retrospective 1980s	219	Stage I-IV	All	6.8	NA (8 years)		No	35 (16-NA)	17 international centers. Treated in 1980s. MOPP based therapy. Compared NLPHL vs lymphocyte rich variant.
			I			85%	99			
			II			71%	94			
			III			62%	94			
			IV			24%	41			
Wirth et al. (2005) [21]	Retrospective 1969–1995	202	Stage I–II	RT	15	82% (15 years)	83% (15 years)	Yes	31 (2–79)	Australian. Age > 45 years, B symptoms, and number of involved sites with inferior OS.
Nogova´ et al. (2008) [18]	Retrospective	394	Stage I-IV	All	50 m	88%	96%	No	37 (16–75)	GHSG HD12 and HD14. Inferior PFS with advanced stage, low Hb and lymphopenia. Study to compare NLPHL vs cHL, showed better outcome for NLPHL than cHL
			early Favor			93%				
			early unfav			87%				
			advanced			77%				
Chen et al. (2010) [22]	Retrospective 70–2005	113	Stage I–II	RT, CMT, CT	136	at 10 years	at 10 years	Yes	27 (3–77)	From Boston, USA. RT 82% pts., CMT 12% and CT alone 6%. PFS limited RT 64%, regional RT 84%, EFRT 81%. Chemo + RT with inferior OS.
			Stage I			85%	96%			
			Stage II			61%	100%			
Biasoli et al. (2010) [3]	Retrospective (1973–2003)	164	Stage I-IV		9.5 yrs	60% (10 yrs)	91% (10 yrs)	Yes	30 (6–69)	French centers, 158 stage I-II. 11.6% had transformation
				any Rx		66%	93%			
				No Rx		41%	91%			
Solanki et al. 2013 [23]	Retrospective 1988–2009	469	Stage I-II	NA	6 yrs	NA	88%	No	37 [4–88]	SEER data USA. older age, female gender, multiple nodal regions and B-symptoms with inferior OS.
Eichenauer et al. (2015) [24]	Retrospective 1988–2009	256	Stage IA		91 m	88.9% (8 years)	98.2 (8 years)	No	39 (16–75)	GHSG 1988–2009 studies. All have similar tumor control. IFRT with less toxicity.
		72		CMT	95	88.5% (8 years)	98.6% (8 years)			
		49		EFRT	110	84.3% (8 years)	95.7% (8 years)			
		108		IFRT	87	91.9% (8 years)	99.0% (8 years)			
		27		Rituximab	49	81% (4 years)	100% (4 years)			
Gerber et al. 2015 [25]	Retrospective 1988–2010	1162	Stage I-IV	NA	7 yrs	NA	91% & 83% (5–10 yrs)	No	38 (not available)	SEER data USA. A Population-Based analysis on NLPHL vs cHL. No info related to chemo.
Lazarovici et al. (2015) [5]	Retrospective (1974–2012)	314	Stage I-IV	All	55.8 m	44.2% (10 yrs)	94.9% (10 yrs)	Yes	38 (18–79)	LYSA (France + Belgium). 82.5 stages I-II. Transformation 7.6%
				CT		78.8% (4 yrs)				
				RT		79.6% (4 yrs)				
				CMT		93.9% (4 yrs)				
Parikh et al. 2016 [26]	Retrospective 1998–2011	1016	Stage I-II		6.6 yrs	NA		No	44 (18–90)	National Data Base (USA). Exploring the impact of RT, showed that RT use with better OS.
				No RT			88.10%			
				Yes RT			94.10%			
Kenderian et al. 2016 [8]	Retrospective 1970–2011	222	Stage I-IV		16.3 yrs	NA		No	40 (15–81)	Mayo Clinic. Focused on HGT, 8.1% HGT at a median of 35 months, 76% 5 yr OS. No difference in OS of HGT vs no HGT NLPHL pts.
			HGT				76.4 (5 yrs)			
			No HGT				similar as transformed			
Molin et al. (2017) [27]	Retrospective (2000–2014)	158	Stage I-IV	All	7.3 yrs	NA	94 and 79% (5-10 yrs)	No	48 (16–88) male 44 female 59	Swedish Cancer Registry. No survival benefit of RT vs CMT in stages I-IIA. Rituximab with chemo superior to chemo alone in IIB-IV patients.
			Stages I-IIA				98 and 85%			
			stages IIB-IV				87 and 67%			
Shivarov 2018 [28]	Retrospective (2000–2014)	1401	Stage I-IV		NA	NA			43 (16–92) male 41 female 48	SEER data USA. OS extracted from the figure. Older age, stage III-IV and not using RT with inferior OS. Female older at Dx.
			I-II				90 approx			
			III-IV				75 approx			
Alonso et al. (2018) [29]	Retrospective (2004–2012)	1420	Stage I-II		48.3 m	NA	(10 years)	No	45 RT gp, 48 No RT gp	National Data Base (USA). Age > 60 and not using RT with inferior OS.
				No Rx			87%		48	
				CT			80%		48	
				RT			93%		45	
				CMT			92%		45	
Posthuma et al. (2019) [19]	Retrospective (1993–2016)	687	Stage I-IV	All	7.7 yrs	NA	(5–10 yrs)	No	4–87	Netherland Cancer Registry. Increase in median age over time. No excess mortality compared to general population in pts. age 18–39.
			Stage I-II	No Rx			93–80%		42 (2003–2016)	
				RT			99–99%			
				Chemo +/− RT			90–89%			
			stage III-IV	No Rx			61–57%			
				Chemo +/− RT			89–80%			
Borchmann et al. (2019) [4]	Retrospective (1974–2016)	163	Stage I-IV	All	69 m	85% (5 yrs)	99% (5 yrs)		40 (16–75)	Memorial Sloan Kettering USA, transformation 7.4% at 7 years median. Shorter PFS with extranodal and bulky disease > 5 cm. Non RT Rx with inferior PFS
				No Rx		77%	100%	Yes		
				any Rx		87%	98%			
			Early stage	No Rx		65%				
				any Rx		94%				
				With RT		94%				
				without RT		73%				
Eichenauer et al 2019 [6]	Retrospective	471	Stage I-IV		9.2 yrs	75.5%	92.10%		39 (16–75)	GHSG HD7 to HD15. HGT 2.54%. 23% of all deaths due to NLPHL
			early Favor			79.7%	93.30%			
			early unfav			72.1%	96.20%			
			advanced			69.8%	87.40%			
Wilson et al. 2019 [7]	Retrospective (2004–2014)	233	Stage I-IV	All	8.6	88% (estimated)	97% (5 yrs)	No	43 (< 16 only 5%)	Multi-institution from UK. HGT 6%.
				No Rx			96.70%			
				RT			98.3			
				RT +/− chemo			95.2			
Current study	Retrospective 1998–2019	200	Stage I-IV	All	5	62.3% & 54% [5–10]	94.8% & 92.4 (5–10)	Yes	22 (4–79)	Single institution. Chemo + RT with better EFS than chemo alone. High incidence of familial malignancies, large numbers with HDC auto-SCT,
			I			72% (10 yrs)	97.7			
			II			55.7%	97.7			
			III			20%	87.6			
			IV			26.8%	79.5			

*Abbreviations*: Same as Tables 1 and 2, *PFS* Progression free survival, *EFS* Event free survival, *Rx* Treatment, *GHSG* German Hodgkin Lymphoma Study Group, *SEER* Surveillance, Epidemiology, and End Results, *CMT* Combined modality treatment, *EFRT* Extended field RT, *IFRT* Involved field RT, *HGT* High grade transformation

Like most of the studies, we have observed the same gender distribution of Male:female of 3:1. The median age of our patients at diagnosis is 22 years with half of the patients < 21 years at diagnosis. Even for patients > 14 years, our median age is 25.7 years (14–79 years). This is significantly different from other reports (Table 4). This is a reflection of the significantly large young population in the Kingdom of Saudi Arabia (as well as in the Middle East) as reported before [30]. Given the relatively small number of females and different age distribution as stated above, we failed to observe any EFS difference due to gender and age at diagnosis as shown in some other studies (Table 4). We also failed to show the negative impact of older age on various survival outcomes due to the same reason / limited older population.

We observed the same stage distribution as others, almost > 3/4 with stages I-II. Despite the increasing use of FDG-PET/CT scan from 2010 to 2019 for initial staging, we observed no change in the staging trend from the earlier period.

We treated our patients according to uniform guidelines that are updated frequently. NLPHL was treated as classical Hodgkin lymphoma (cHL) protocols. ABVD was the mainstay of chemotherapy (87%), although, for the past few years, we are increasingly using the non-anthracycline-based treatment for early stages, i.e. rituximab + CVP or a CHOP based treatment for advanced stages. As the treatment and survival outcomes are different for stage I-II vs III-IV, we have reported them separately. Patients with stages I-II were mainly treated with chemotherapy + RT. This is relatively different from many studies where mostly RT alone was used as the primary treatment modality. One of the main reasons was to minimize/avoid radiation in our young patient population. Overall survival outcome is similar for this group regardless of treatment arm, although the use of RT resulted in superior EFS compared to chemotherapy alone group. We also reported differences in the outcome of stage I-II patients who received RT according to the reason for the use of RT. RT use showed a 5-year EFS of 97% in patients who were already in CR after chemotherapy vs 63% EFS when used as primary/first-line treatment vs 36% for the eradication of residual disease after chemotherapy. For stages III-IV, due to relatively small numbers of uniformly treated patients (chemotherapy-based), no subgroup analysis was performed. Rituximab alone, or in combination with ABVD was not used in the primary/salvage treatment according to the approved treatment guidelines at that time. Unexpected pulmonary toxicity resulted in the death of two patients in CR on Rituximab + ABVD resulting in the reluctance to use rituximab. The impact of rituximab in our patient population should be interpreted with caution. There are a wide variety of clinical scenarios, timing, type, and intensity of salvage and all these have a significant impact on the outcome. Given the small number of patients in each rituximab-receiving group in our cohort, the magnitude of true benefit remains unclear. At the same time, clinical trials are unlikely to be conducted in this setup due to the safety and efficacy of rituximab. In the salvage setup, we used ESHAP, lately with rituximab as our primary salvage chemotherapy for patients going for HDC auto-SCT [9]. We previously reported seventeen patients who underwent HDC auto-SCT data and now total HDC auto-SCT patients have reached twenty-nine [9]. This is one of the largest single-institution experiences of NLPHL and HDC auto-SCT that proves the benefit of this strategy for patients with refractory NLPHL. Detailed information on multiple lines of treatment makes our data a nice reference.

We also tried to explore the impact of various factors using multivariate analysis. For stages I-II, we identified GHSH group of early favorable and early unfavorable as discriminatory for the EFS. For stages III-IV, hemoglobin and albumin levels were significant and we were able to draw a risk-based EFS graph. Given the small numbers, this should be taken with caution. We also observed that HL- International Prognostic Score based model for EFS has the same outcome and most likely will a better choice to validate in a larger group of stages III-IV.

We reported 5.5% HGT, this is in line with many other studies in Table 3 (6 to 11.6%), except for one GHSG study with a lower incidence of 2.45% HGT6. These HGT patients have excellent OS as reported in other studies. Most of our patients with HGT received HDC auto-SCT. Longer follow-up is needed for those patients who have relatively short follow-up / less than 5 years of follow-up for an accurate incidence of HGT.

A quarter of our patients had some form of pathologically proven reactive lymphadenopathy at some point in time. In the era of FDG-PET/CT scan, these findings are likely to be more and a change in the treatment strategy should be made only after a pathological confirmation. This is especially true when HGT is also a concern in NLPHL. There are reports with a limited number of patients with this observation [31–34].

## Conclusion

In brief, our data has shown that NLPHL presentation has both similarities and differences in the Middle East. Younger age at the presentation in our patients has its impact on treatment decisions and likely long-term survival. FDG-PET/CT scan has not resulted in stage migration. For early stages, various treatment strategies have no impact on overall survival, though treatment failure was less common for those who received chemotherapy + RT compared to chemotherapy alone. A large number of patients had FHM that warrants genetic studies. Lymphoid hyperplasia may mimic a relapse and proper pathological confirmation is important before changing a treatment plan.

## Data Availability

All authors had full access to all data and approved this submission. The datasets used and/or analyzed during the current study are available from the corresponding author on reasonable request. Data sharing: Data is a “retrospective/prospective database” and is owned by the institution. This data is under the custody of the principal and co-investigators as per the Institutional Research Advisory Counsel and Research Ethics Committee guidelines. It is not available to the public for sharing. Full or part of this data can be shared after an official request / approval of the institutional Research Advisory Counsel and Research Ethics Committee.

## Abbreviations

NLPHL: Nodular lymphocyte-predominant Hodgkin lymphoma
KM: Kaplan-Meier
RT: Radiation therapy
ABVD: Adriamycin, bleomycin, vinblastin, dacarbazine
OS: Overall survival
EFS: Event free survival
HL: Hodgkin lymphoma
LP cells: Lymphocyte-predominant cells”
NHL: Non-Hodgkin lymphoma
DLBCL: Diffuse large B cell lymphoma
HDC auto-SCT: High dose chemotherapy and auto-SCT
HGT: High-grade transformation
PR: Partial response
SD: No response (NR)/ stable disease
PD: Progressive disease
CR: Complete remission
CRu: CR unconfirmed
EFRT: Extended field RT
IFRT: Involved field RT
ISRT: Involved site RT
VMAT: Volumetric modulated arc therapy
IMRT: Intensity-modulated RT
FDG-PET/CT: ^18^F-fluorodeoxyglucose positron emission tomography
WHO: The World Health Organization
GHSG: German Hodgkin Study Group
COPP/MOPP: Cyclophosphamide / mechlorethamine, vincristine, procarbazine, prednisone
R-CHOP: Rituximab + cyclophosphamide, adriamycin, vincristine, prednisone
CVP: Cyclophosphamide, vincristine, prednisone
HR: Hazard ratio
CI: Confidence interval
cHL: Classical Hodgkin lymphoma
SEER: Surveillance, Epidemiology, and End Results
CMT: Combined modality treatment
